# Inhibition of PI3K/Akt/mTOR overcomes cisplatin resistance in the triple negative breast cancer cell line HCC38

**DOI:** 10.1186/s12885-017-3695-5

**Published:** 2017-11-03

**Authors:** Katharina Gohr, Alexandra Hamacher, Laura H. Engelke, Matthias U. Kassack

**Affiliations:** Institute for Pharmaceutical and Medicinal Chemistry, Heinrich Heine University Düsseldorf, Universitätsstraße 1, 40225 Düsseldorf, Germany

**Keywords:** Triple negative breast cancer, HCC38, MDA-MB231, EGFR, IGF1R, NVP-AEW541, NVP-BEZ235, Lapatinib, Cisplatin resistance

## Abstract

**Background:**

Widely established targeted therapies directed at triple negative breast cancer (TNBC) are missing. Classical chemotherapy remains the systemic treatment option. Cisplatin has been tested in TNBC but bears the disadvantage of resistance development. The purpose of this study was to identify resistance mechanisms in cisplatin-resistant TNBC cell lines and select targeted therapies based on these findings.

**Methods:**

The TNBC cell lines HCC38 and MDA-MB231 were subjected to intermittent cisplatin treatment resulting in the 3.5-fold cisplatin-resistant subclone HCC38CisR and the 2.1-fold more resistant MDA-MB231CisR. Activation of pro-survival pathways was explored by immunostaining of phospho-receptor tyrosine kinases. Targeted therapies (NVP-AEW541, lapatinib and NVP-BEZ235) against activated pathways were investigated regarding cancer cell growth and cisplatin sensitivity.

**Results:**

In HCC38CisR and MDA-MB231CisR, phosphorylation of epidermal growth factor receptor (EGFR) and insulin-like growth factor 1 receptor (IGF1R) was observed. In HCC38CisR, treatment with NVP-AEW541 increased potency of lapatinib almost seven-fold, but both compounds could not restore cisplatin sensitivity. However, the dual phosphoinositide 3-kinase (PI3K) and mammalian target of rapamycin (mTOR) inhibitor NVP-BEZ235 acted synergistically with cisplatin in HCC38CisR and fully restored cisplatin sensitivity. Similarly, NVP-BEZ235 increased cisplatin potency in MDA-MB231CisR. Furthermore, NVP-AEW541 in combination with lapatinib restored cisplatin sensitivity in MDA-MB231CisR.

**Conclusion:**

Simultaneous inhibition of EGFR and IGF1R in cisplatin-resistant TNBC cell lines was synergistic regarding inhibition of proliferation and induction of apoptosis. Co-treatment with NVP-BEZ235 or with a combination of NVP-AEW541 and lapatinib restored cisplatin sensitivity and may constitute a targeted treatment option for cisplatin-resistant TNBC.

**Electronic supplementary material:**

The online version of this article (10.1186/s12885-017-3695-5) contains supplementary material, which is available to authorized users.

## Background

Breast cancer is the second most common cancer in the world and the incidence of female breast cancer has continuously increased [1]. In 2013, 1.8 million incident cases of breast cancer occurred, and the disease caused 464,000 deaths [1]. Triple negative breast cancer (TNBC) accounts for 10–20% of these breast cancer cases [2]. This type of breast cancer is defined by lacking protein expression of progesterone (PR) and estrogen receptors (ER) as well as by low ErbB2 expression. For this reason, TNBCs cannot benefit from endocrine therapies or trastuzumab [3]. Therefore, chemotherapy is the systemic treatment option. The use of cisplatin and carboplatin in treatment of TNBCs is currently investigated in clinical trials and initial results indicate a beneficial effect for cisplatin in neoadjuvant chemotherapy [4, 5]. One major challenge in cisplatin therapy is drug resistance which can be intrinsic or occur after several cycles of therapy. Trigger for cisplatin resistance can be found pre-target (e.g. reduced uptake), on-target (e.g. increased DNA-repair), post-target (e.g. inactivation of TP53) or off-target [6]. Off-target mechanisms include activation of pro-survival pathways mediated for example via growth factor receptors.

We have previously shown that resveratrol or ellagic acid prevented the development of cisplatin resistance in the ovarian cancer cell line A2780. This effect is at least in part based on the prevention of activation of ErbB2 and ErbB3 in the course of long-term cisplatin treatment [7]. IGF1R activation has also been shown to be a crucial step in the development of cisplatin resistance [8]. Activation of growth factor receptors may also play a role in the development of cisplatin resistance in TNBC and due to their involvement in cell proliferation, apoptosis and metastasis they are considered attractive targets for therapies beyond classical chemotherapeutic drugs [9]. In 1998, a link between elevated insulin-like growth factor 1 (IGF1) blood levels and breast cancer risk in premenopausal women has been published [10]. In this context the IGF1R emerged as a promising target in cancer therapy. Binding of its ligands to IGF1R results in the activation of mainly two downstream signaling networks: PI3K-Akt-mTOR and RAF-MAPK, both linked to cell survival and inhibition of apoptosis. Interestingly, not high expression but high phosphorylation of IGF1R was predictive for poor prognosis in breast cancer [11]. Extensive research in this area was done but after initially promising results, phase III clinical trials using anti-IGF1R-targeted therapies were mainly disappointing [12]. These findings might be due to resistance mechanisms like compensatory signaling via growth hormone receptors, insulin receptors or epidermal growth factor receptors. Therefore, combination therapies were suggested. In vitro studies showed a synergistic effect of a small molecule IGF1R inhibitor with gefitinib as EGFR/ErbB2 inhibitor [13]. However, as has been seen for IGF1R inhibitors alone, larger clinical trials combining IGF1R inhibitors with either gefitinib or erlotinib failed [14]. Taking into account that no biomarkers were used to predict response, predictive tools for the use of IGF1R inhibitors might be necessary.

The purpose of our study was to identify resistance mechanisms in a cisplatin-resistant TNBC cell line leading to targeted therapies as treatment options in this cancer type. Evaluation of the phosphorylation status of receptor tyrosine kinases revealed activation of IGF1R and EGFR as a result of cisplatin resistance. Therefore, inhibitors of these two receptors (NVP-AEW541 and lapatinib) and an inhibitor of downstream acting PI3K/Akt/mTOR (NVP-BEZ235) were evaluated regarding their effects on cancer cell growth and cisplatin sensitivity. Indeed, co-treatment of NVP-AEW541 with lapatinib increased potency of lapatinib in the cisplatin-resistant TNBC cell line HCC38CisR but did not increase cisplatin sensitivity. On the other hand, NVP-BEZ235 acted synergistically with cisplatin and fully restored cisplatin sensitivity in HCC38CisR. Furthermore, in the highly cisplatin-resistant TNBC cell line MDA-MB231CisR, treatment with NVP-BEZ235 or co-treatment of NVP-AEW541 with lapatinib increased potency of cisplatin up to 4.8-fold.

## Methods

### Materials

NVP-AEW541 and NVP-BEZ235 were gifts from Novartis (Basel, Switzerland). Lapatinib, KU0063794 and LY294002 were from Cayman Chemical (Michigan, USA). Cisplatin was purchased from Sigma-Aldrich (Steinheim, Germany). 3-(4,5-Dimethylthiazol-2-yl)-2,5-diphenyltetrazolium bromide (MTT) was purchased from Serva (Heidelberg, Germany). Propidium iodide was from PromoCell (Heidelberg, Germany). Roswell Park Memorial Institute (RPMI) media 1640, Dulbecco’s Modified Eagle Medium (DMEM), fetal bovine serum (FBS), penicillin/streptomycin [10,000 U/ml; 10 mg/ml] and trypsin-EDTA (0.05% trypsin, 0.02% EDTA in PBS) were purchased from PAN Biotech (Aidenbach, Germany). Primary antibodies were purchased from R&D Systems (Wiesbaden, Germany) (pIGF1R, IGF1R, p-EGFR, EGFR, p-ErbB2, ErbB2, p-ErbB3, ErbB3) or Santa Cruz Biotechnology (Heidelberg, Germany) (p-Akt, Akt, β-Actin, PARP). HRP-conjugated secondary antibodies were from R&D Systems. All other reagents and chemicals were from VWR BDH PROLABO (Darmstadt, Germany).

### Cell lines and cell culture

The triple negative breast cancer cell line HCC38 was obtained from ATCC (Manassas, USA, ATCC order number: ATCC® CRL-2314™) and cultivated in RPMI-1640 medium supplemented with 10% FBS, 120 μg/ml streptomycin and 120 U/ml penicillin. The TNBC cell line MDA-MB231 (ATCC, Manassas, USA, ATCC order number: ATCC® HTB-26™) was cultivated in DMEM supplemented with 15% FBS, 120 μg/ml streptomycin and 120 U/ml penicillin. Cells were grown at 37 °C in a humidified atmosphere containing 5% CO_2_. HCC38CisR and MDA-MB231CisR, the cisplatin resistant subclones of HCC38 and MDA-MB231, respectively, were generated by intermittent treatment of HCC38 or MDA-MB231 with cisplatin for 40 cycles according to methods previously published [7, 8, 15]. Cells were grown to 80–90% confluency before using them for assays.

### MTT cell viability assay

Cell viability was determined using the MTT assay as previously described [7]. Resistance factor was calculated as ratio of IC_50_ of the resistant cell line and IC_50_ of the sensitive cell line. To investigate the effect of the small molecule inhibitors on cisplatin cytotoxicity, compounds were added 48 h prior to 72 h cisplatin treatment. For combination index analysis, cell viability was determined from each well relative to the average absorbance of control wells. The combination indexes (CIs) were calculated using CalcuSyn 2.1 software (Biosoft, Cambridge, U.K.) based on the Chou − Talalay method [16]. CI > 1 indicates antagonism. CI = 1 indicates an additive effect and CI < 0.9 indicates synergism.

### Neutral red cell viability assay

To exclude compound effects potentially influencing mitochondrial activity, neutral red cell viability assay instead of MTT assay was performed as previously described [17]. Briefly, after incubation time, medium was removed and 200 μl neutral red incubation solution (medium containing FBS, 0.1 M HEPES buffer pH 7.4 and 0.01% neutral red) was added. After 2 h, incubation solution was removed and cells were quickly washed with 1% CaCl_2_ × 2 H_2_O in 1% formaldehyde solution. After a second washing step, cells were lysed with a 1:1 mixture of ethanol and 1% acetic acid. Absorbance was measured at 544 and 690 nm in a FLUOstar microplate reader (BMG Labtech, Ortenberg, Germany).

### Doubling time

The assay was performed as previously described [7]. Cells were seeded in 6-well plates (Sarstedt AG, Nürmbrecht, Germany). After 24, 48, 72, and 96 h, cells were trypsinized and washed with PBS. Total number of cells in 1 ml buffer was counted in a CyFlow® space (Partec, Muenster, Germany). Doubling time was calculated using GraphPad Prism (version 4, GraphPad Software Inc., San Diego, USA).

### Western blotting

For western blotting, standard procedures were used as previously described [7].

### RTK signal pathway analysis

The tyrosine-kinase phospho-proteom was investigated by a human phospho-receptor tyrosine kinase antibody array (Cat# ARY001) from R&D Systems according to the manufacturer’s protocol. Cell lysate containing 300 μg protein was used.

### Cell cycle analysis

Distribution of cell cycle phases of the different cell lines was analyzed by flow cytometry using standard procedures as previously described [7].

### Apoptosis analysis

Apoptotic cells were determined by propidium iodide staining as previously described [7].

### Scratch assay

Scratch assay was performed according to standard procedures as previously described [7]. Cell-free area was determined using ImageJ [18]. Percentage of space that was occupied with cells after 24 h was calculated.

### Statistical analysis

Assays were performed at least in three independent experiments. Concentration effect curves were then generated by nonlinear regression curve fitting using the 4-parameter logistic equation with variable hill slope (GraphPad Prism version 4, GraphPad Software Inc.). Data presented are mean ± SEM if not otherwise stated. Statistical significance was assessed by two-tailed Student’s t-test or ANOVA and considered significant if *p* < 0.05. pIC_50_ ± SEM leading to the reported IC_50_ values are shown in Additional file 1.

## Results

**Fig. 1:**
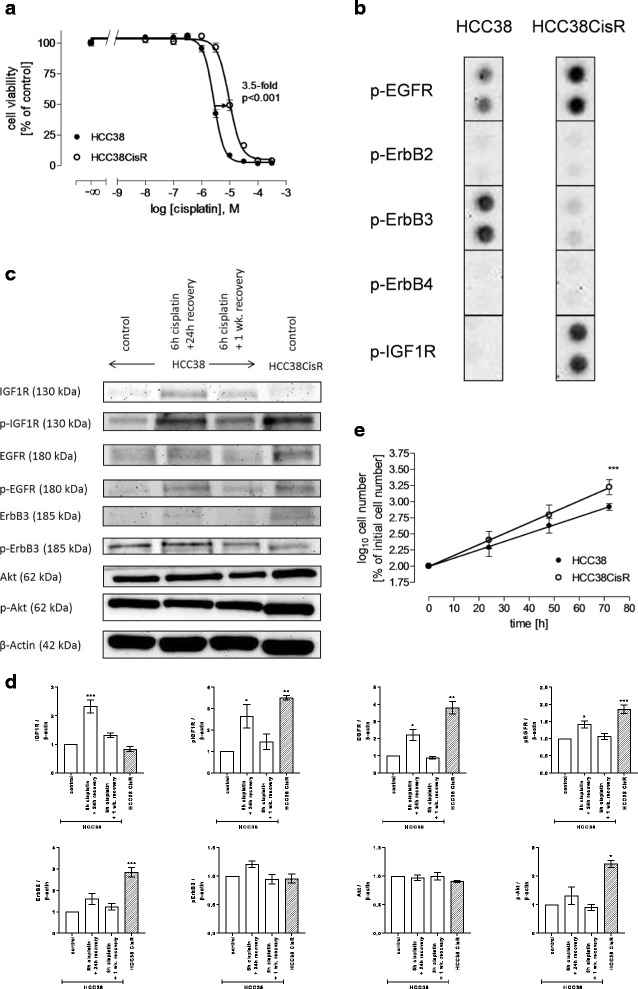
Characterization of HCC38 and cisplatin-resistant HCC38CisR. **(A)** Weekly exposure of HCC38 with the IC_50_ of cisplatin for 6 h resulted in the cisplatin resistant subclone HCC38CisR with a resistance factor of at least 3.5 (*p* < 0.001). IC_50_ cisplatin HCC38: 2.7 μM; IC_50_ cisplatin HCC38CisR: 9.4 μM. Shown are mean +/− SEM, *n* = 3. **b** Detail of phospho-RTK-array displays phosphorylation status of EGFR-family and IGF1R in HCC38 and HCC38CisR. **c** Immunostaining of expression and activation of signaling kinases. Shown is a representative experiment out of 3. HCC38 cells were treated with 2.5 μM cisplatin for 6 h followed by a recovery of 24 h or 1 week. Untreated HCC38 and HCC38CisR served as controls. **d** Densitometric analysis of the protein bands of HCC38 and HCC38CisR were performed using ImageJ software (NIH). Data are means ± SD, *n* = 3. All values have been normalized to HCC38 control. Statistical analysis was performed using one-way ANOVA test (* *p* < 0.05, ** *p* < 0.01, and *** p < 0.001). **e** Cell proliferation measured by flow cytometry-based cell counting. Doubling times were 23.6 h in HCC38 and 16.9 h in HCC38CisR and were significantly different (*** *p* < 0.001). Shown are mean +/− SEM, *n* = 3

The cisplatin resistant cell line HCC38CisR was generated by weekly exposure to the IC_50_ of cisplatin for 6 h. After 40 cycles, the IC_50_ (determined by MTT) has shifted from 2.7 μM to 9.4 μM corresponding to a resistance factor of 3.5 (Fig. 1a). This resistance factor is in the range of previously reported resistance factors of cell lines established from cancer patients before and after chemotherapy [19]. Resistance could be maintained without further cisplatin treatment. IC_50_ of cisplatin varied throughout the duration of these studies between 7 and 12 μM for HCC38CisR. HCC38CisR was characterized in comparison to the parental cell line HCC38. Phospho-receptor tyrosine kinase antibody array was used to determine receptor activation. Other receptors than those shown in Fig. 1b (EGFR-family, IGF1R) were not differentially phosphorylated. HCC38 showed – as expected – no activation of ErbB2 but activation of EGFR and ErbB3. Cisplatin resistance (HCC38CisR) did not generate ErbB2 activation, while EGFR and IGF1R showed a markedly enhanced activation in HCC38CisR (Fig. 1b). In contrast, ErbB3 activation was diminished in HCC38CisR. These results could be confirmed by western blotting (Fig. 1c and d). In this assay, expression and activation of receptor tyrosine kinases (RTKs) was estimated in HCC38, HCC38CisR (long-term cisplatin stress, 40× intermittent 6 h cisplatin treatment), and HCC38 exposed to short-term cisplatin stress (6 h IC_50_ of cisplatin with 24 h or 1 week recovery). IGF1R and EGFR phosphorylation was increased after 6 h cisplatin stress and 24 h recovery, and in HCC38CisR. If HCC38 treated 6 h with cisplatin could recover from cisplatin stress for one week, receptor phosphorylation decreased nearly to the initial state. Evaluating the expression of growth factor receptors, there was hardly any difference between untreated HCC38 and HCC38CisR. Akt expression and phosphorylation was enhanced in HCC38CisR compared to HCC38 either untreated or short-term treated with cisplatin. Long-term cisplatin treatment resulting in HCC38CisR further increased proliferation rate and decreased doubling time significantly from 24 h to 17 h as displayed in Fig. 1e.

**Fig. 2:**
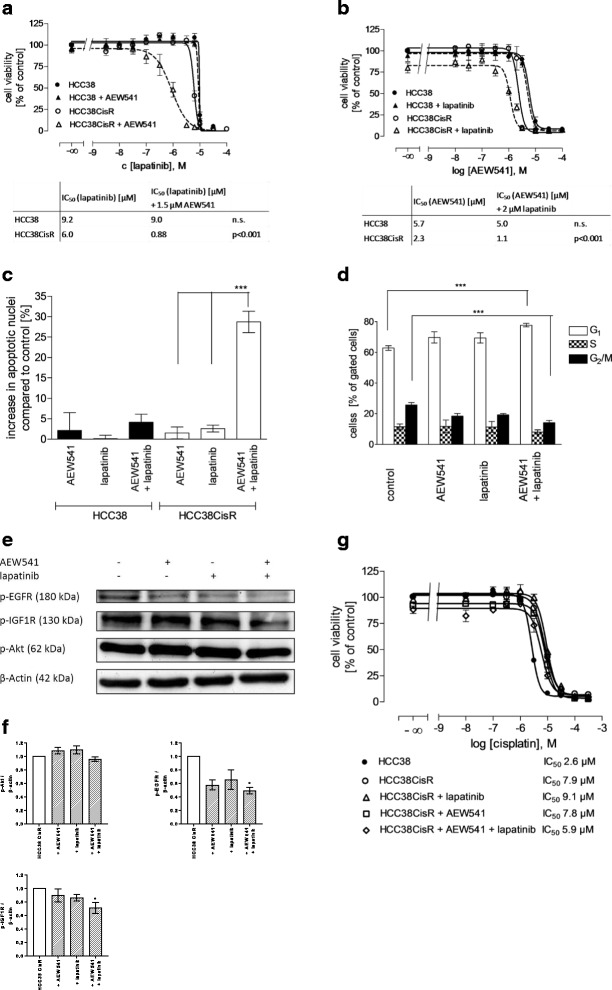
Combination of lapatinib and NVP-AEW541 is hyper-additive but not reversing cisplatin resistance in HCC38CisR. **a** Coincubation with 1.5 μM NVP-AEW541 significantly decreased IC_50_ of lapatinib in HCC38CisR, whereas this treatment had no effect in HCC38. **b** Coincubation with 2 μM lapatinib significantly decreased IC_50_ of NVP-AEW541 in HCC38CisR but had no effect in HCC38. **c** In HCC38CisR (but not in HCC38), the combination of NVP-AEW541 and lapatinib significantly induced apoptosis in a hyper-additive manner (***p < 0.001). NVP-AEW541 and lapatinib were used at 2 μM. Cells were treated for 48 h and the amount of apoptotic nuclei in the control was subtracted from treated samples. **d** Effect of NVP-AEW541 or lapatinib (2 μM, respectively) on cell cycle in HCC38CisR. Combination of 2 μM NVP-AEW541 and 2 μM lapatinib significantly (***p < 0.001) increased cell population in G_1_ (77.7 ± 1.2% vs. 67.3 ± 1.4%) while reducing cell population in G_2_/M phase (14.2 ± 1.5% vs. 25.7 ± 1.6%). Incubation time was 48 h. **e** Western blot analysis of p-EGFR, p-IGF1R, and p-Akt upon treatment of HCC38CisR with an IC_50_ of lapatinib or NVP-AEW541 or both compounds for 6 h. **f** Densitometric analysis of the protein bands for p-AKT, p-EGFR, and p-IGF1R of HCC38CisR were performed using ImageJ software (NIH). Data are means ± SD, *n* = 3. All values have been normalized to untreated HCC38 CisR. Statistical analysis was performed using one-way ANOVA test (* *p* < 0.05). **g** Effect of 1 μM lapatinib and 1.5 μM NVP-AEW541 on cisplatin sensitivity either alone or in combination. Lapatinib and/or NVP-AEW541 were added 48 h prior to cisplatin treatment. IC_50_ of cisplatin did not significantly differ. All data shown are mean +/− SEM, *n* = 3, except (**e**) showing a representative experiment out of 3

Based on activation of EGFR and IGF1R in HCC38CisR (Fig. 1), the dual EGFR/ErbB2 inhibitor lapatinib and the IGF1R inhibitor NVP-AEW541 were chosen for further experiments. The IC_50_ of both inhibitors was lower in HCC38CisR than in HCC38 (Fig. 2a/b). The effect was more pronounced for NVP-AEW541 (5.7 μM vs. 2.3 μM) than for lapatinib (9.2 μM vs. 6.0 μM) (Fig. 2a/b). Next, we tested the combination of both inhibitors. In HCC38, co-incubation of NVP-AEW541 had no effect on the IC_50_ of lapatinib, and vice versa, coincubation of lapatinib had no effect on the IC_50_ of NVP-AEW541 (Fig. 2a/b). However, coincubation of NVP-AEW541 caused a significant increase in potency of lapatinib in HCC38CisR (almost 7-fold from 6.0 to 0.88 μM, Fig. 2a). Vice-versa, coincubation of lapatinib resulted in a significantly decreased IC_50_ for NVP-AEW541 in HCC38CisR (2-fold from 2.3 to 1.1 μM, Fig. 2b). To confirm the observed effects, synergism studies were performed (Table 1). Analysis based on the Chou-Talalay method [16] suggested a synergistic interaction between lapatinib and NVP-AEW541 (combination indexes CI < 0.9) in HCC38CisR.

**Table 1:** Synergism studies between NVP-AEW541 and lapatinib

Lapatinib [μM]					
AEW541 [μM]	1	2	2.5	3	3.5
0.5	^a^	^a^	0.76	0.52	0.46
1	^a^	0.58	0.51	0.44	0.42
1.5	0.96	0.59	0.58	0.51	0.49
2	0.46	0.42	0.46	0.49	0.52
3	0.69	0.57	0.56	0.68	0.64

^a^ fraction affected <0.2

Since MTT assay cannot distinguish between inhibition of proliferation and induction of apoptosis, we examined induction of apoptosis using propidium iodide nuclear staining (Fig. 2c). Both inhibitors were added alone or in combination for 48 h in a concentration of 2 μM. In HCC38 the treatment induced nearly no apoptotic cells (Fig. 2c). In HCC38CisR, NVP-AEW541 (1.53 ± 1.42%) and lapatinib (2.59 ± 0.83%) showed similarly nearly no induction of apoptosis whereas the combination of both compounds could heavily induce apoptosis (28.7 ± 2.62%, Fig. 2c). The effect of this combination on cell cycle distribution in HCC38CisR was then determined using propidium iodide staining (Fig. 2d). Again, NVP-AEW541 and lapatinib alone or in combination were added in a concentration of 2 μM for 48 h prior to ethanol fixation. NVP-AEW541 and lapatinib alone had no significant effects. In contrast, the combination of both compounds could reduce the fraction of cells in the G_2_/M phase from 25.7% to 14.2% while increasing the fraction of cells in G_1_ phase from 62.8% to 77.7% (*p* < 0.001; Fig. 2d). Treatment with lapatinib, NVP-AEW541 or their combination had no effect on cell cycle distribution in HCC38 (see Additional file 2). Next, the effect on phosphorylation of Akt, EGFR and IGF1R after 6 h treatment of HCC38CisR with an IC_50_ of lapatinib or NVP-AEW541 alone or in combination was determined by western blotting (Fig. 2e, f). Whereas both compounds alone had only moderate effects on receptor phosphorylation, their combination reduced EGFR and IGF1R phosphorylation to a greater extent. Interestingly, Akt phosphorylation was unaffected by either treatment.

Since EGFR and IGF1R were activated in cisplatin-resistant HCC38CisR, we examined if the combination of lapatinib and NVP-AEW541 could restore cisplatin sensitivity in HCC38CisR (Fig. 2g). HCC38CisR was pretreated with the inhibitors 48 h prior to cisplatin treatment. The inhibitors alone and in combination had no significant effect on cisplatin sensitivity. In HCC38, the same was observed: neither lapatinib nor NVP-AEW541 alone nor their combination had an effect on cisplatin sensitivity (see Additional file 3).

**Fig. 3:**
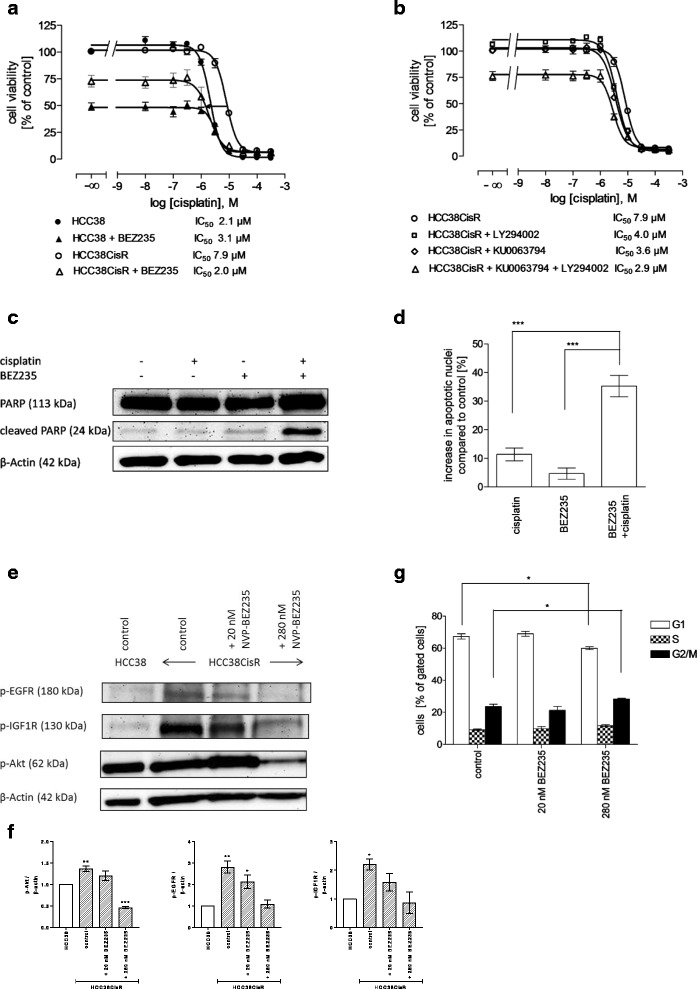
NVP-BEZ235 treatment fully restores cisplatin sensitivity in HCC38CisR. **a** 20 nM NVP-BEZ235 added 48 h prior to cisplatin treatment significantly reduced IC_50_ of cisplatin in HCC38CisR (*p* < 0.001) but not in HCC38. **b** 1 μM KU0063794 or 5 μM LY294002 or their combination significantly reduced IC_50_ of cisplatin in HCC38CisR (p < 0.001). **c** Western blot analysis of PARP and cleaved PARP in HCC38CisR used as an indicator of active Caspase 3. For combination of NVP-BEZ235 and cisplatin, 20 nM NVP-BEZ235 was incubated 48 h prior to addition of 3 μM cisplatin for 6 h. **d** Induction of apoptosis by NVP-BEZ235 and cisplatin. 20 nM NVP-BEZ235 was incubated 24 h prior to addition of 5 μM cisplatin for 6 h followed by 24 h of recovery. Combination of NVP-BEZ235 with cisplatin increased apoptotic nuclei (35.3 ± 3.7%) compared to cisplatin alone (11.4 ± 2.3%) and NVP-BEZ235 alone (4.6 ± 2.0%) (***p < 0.001). **e** Western blot analysis of p-EGFR, p-IGF1R, and p-Akt in HCC38CisR upon 48 h treatment with 20 nM or 280 nM NVP-BEZ235. **f** Densitometric analysis of the protein bands of p-EGFR, p-IGF1R, and p-Akt in HCC38 and HCC38CisR were performed using ImageJ software (NIH). Data are means ± SD, *n* = 3. All values have been normalized to HCC38 control. Statistical analysis was performed using one-way ANOVA test (* *p* < 0.05, ** *p* < 0.01, and *** p < 0.001). **g** Effect of 20 nM or 280 nM NVP-BEZ235 on cell cycle in HCC38CisR. 280 nM NVP-BEZ235 gave a slight but significant (*p < 0.5) reduction of cells in G_1_ phase (67.3 ± 1.6% vs. 60.0 ± 0.9% in control) accompanied by an increase in cells in G_2_/M phase (23.6 ± 1.4% vs. 28.3 ± 0.5% in control). All data shown are mean +/− SEM, n = 3, except (C/E) showing a representative experiment out of 3

It has been shown that cancer cells can easily switch membrane-bound RTK pathways upon inhibition of a particular RTK and still use the same downstream signaling pathways [20]. Further, since neither lapatinib nor NVP-AEW541 had an effect on cisplatin sensitivity and both compounds did not alter Akt phosphorylation increased in HCC38CisR (Fig. 2e, f), we tested whether NVP-BEZ235, a dual inhibitor of PI3K and mTOR, had an effect on cisplatin sensitivity. Evaluating the cytotoxicity of NVP-BEZ235 in HCC38 and HCC38CisR revealed that the IC_50_ was lower in HCC38 (9.1 nM) than in HCC38CisR (69.3 nM) (See Additional file 4). 48 h pretreatment with 20 nM NVP-BEZ235 increased potency of cisplatin in HCC38CisR by a factor of 4 into the range of the non-resistant cell line HCC38 (IC_50_ HCC38CisR: 7.9 μM; IC_50_ HCC38CisR pretreated with 20 nM NVP-BEZ235: 2.0 μM; Fig. 3a). In HCC38, 20 nM NVP-BEZ235 had no effect on cisplatin sensitivity (Fig. 3a). However, NVP-BEZ235 had a more pronounced effect on cell viability in HCC38 as observed by a reduction of the top plateau of the concentration effect curve to 48% in HCC38 versus 74% in HCC38CisR (Fig. 3a). To corroborate the observed effect in HCC38CisR, synergism studies were performed. The calculated CIs indicated synergism between cisplatin and NVP-BEZ235 in HCC38CisR (Table 2). Because NVP-BEZ235 inhibits PI3K as well as mTOR, we examined the effect on cisplatin sensitivity of compounds inhibiting only one of these targets: LY294002 was chosen as PI3K inhibitor, KU0063794 as mTOR inhibitor (Fig. 3b). 48 h preincubation with either compound prior to cisplatin treatment could significantly (*p* < 0.001) sensitize HCC38CisR for cisplatin treatment by a factor of approximately 2. If both inhibitors LY294002 and KU0063794 were combined in 48 h preincubation prior to cisplatin treatment in HCC38CisR, the cisplatin IC_50_ of the parental cell line HCC38 was nearly restored (2.9 μM, Fig. 3b). The effect of NVP-BEZ235 on cisplatin sensitivity was slightly, but significantly (*p* < 0.05) stronger than the effect of the combination of KU0063794 and LY294002.

**Table 2:** Synergism studies between cisplatin and NVP-BEZ235

BEZ235 [nM]					
cisplatin [μM]	30	40	50	60	70
1	^a^	^a^	0.74	0.69	0.76
2	0.76	0.70	0.71	0.60	0.66
3	0.76	0.59	0.61	0.60	0.64
5	0.71	0.63	0.62	0.61	0.67
7	0.70	0.59	0.61	0.60	0.67

^a^fraction affected <0.2

Synergism between NVP-BEZ235 and cisplatin was observed in MTT (Table 2) and further verified by western blotting (Fig. 3c) and apoptosis assay (Fig. 3d). 48 h preincubation with 20 nM NVP-BEZ235 followed by a 6 h treatment with 3 μM cisplatin led to a markedly enhanced accumulation of cleaved poly ADP-ribose polymerase (PARP) in HCC38CisR serving as an indicator of caspase 3 activation. Whereas either compound alone could not induce PARP cleavage, the combination of NVP-BEZ235 and cisplatin markedly induced PARP cleavage. This effect was not observed in HCC38 (see Additional file 5). Similarly NVP-BEZ235 could enhance the number of cisplatin-induced apoptotic nuclei significantly (hyper-additive) without having an own pronounced apoptotic effect. Whereas cisplatin alone caused 11.4% apoptotic nuclei, addition of NVP-BEZ235 tripled this effect (35.3%). Again, this effect could not be observed in HCC38 (see Additional file 6).

Since the effect of NVP-BEZ235 on its different targets is concentration-dependent [21], we tested a low (20 nM) and a high (280 nM) concentration of NVP-BEZ235 on EGFR, IGF1R and Akt phosphorylation (Fig. 3e, f) in HCC38CisR. 280 nM NVP-BEZ235 reduced Akt phosphorylation whereas 20 nM had no effect. Further, phosphorylation of IGF1R and EGFR was diminished, particularly at 280 nM NVP-BEZ235. Cell cycle was only affected by 280 nM (but not 20 nM) NVP-BEZ235 in HCC38CisR (Fig. 3g): cells in G_2_/M phase slightly increased compared to control (28.3% versus 23.6%) accompanied by a slight decrease of cells in G_1_ phase (60.0% versus 67.3%; *p* < 0.05; Fig. 3g).

**Fig. 4:**
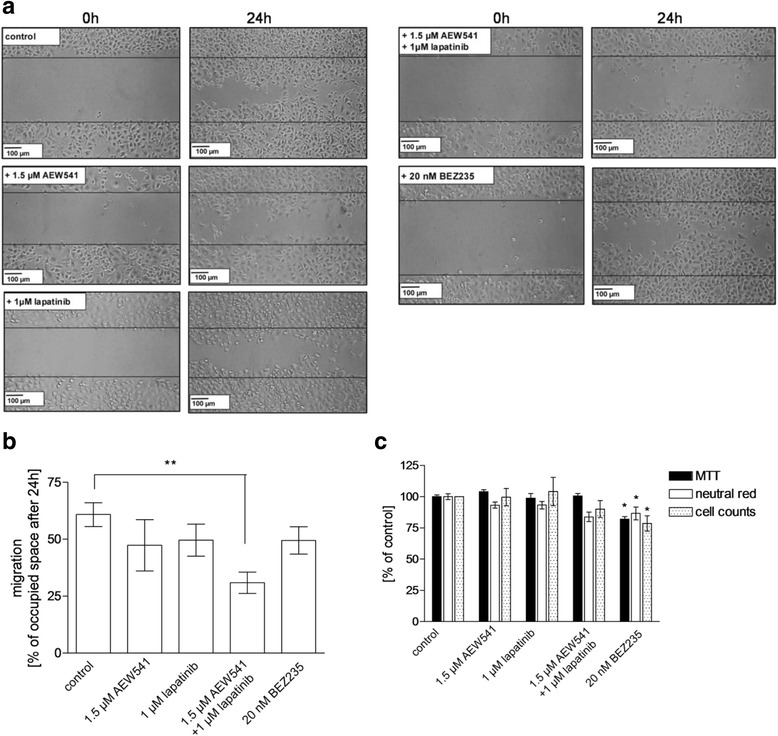
Combination of lapatinib and NVP-AEW541 – but not NVP-BEZ235 inhibits cell migration in HCC38CisR measured by scratch assay. **a** Microscopic images were obtained before (0 h) and 24 h after applying a pipet tip-induced scratch in a nearly confluent cell monolayer. Data shown are one typical experiment out of three independent experiments. **b** Average migration, estimated as space occupied after 24 h, showed that only the combination of 1.5 μM NVP-AEW541 and 1 μM lapatinib significantly reduced cell migration (**p < 0.01). **c** Cell proliferation assays applied under the conditions of (**b)** (24 h incubation). Only 20 nM NVP-BEZ235 significantly reduced cellular proliferation (n = 3, *p < 0.05)

Eventually, we studied effects of the examined kinase inhibitors NVP-AEW541, lapatinib and NVP-BEZ235 on the migratory potential of HCC38CisR by a scratch assay (Fig. 4). 24 h after applying a scratch to untreated cells, 61% of the scratch was covered by cells (Fig. 4a/b). Treatment with any of the kinase inhibitors reduced migration, however only the combination of 1.5 μM NVP-AEW541 and 1 μM lapatinib showed a significant inhibition of migration (Fig. 4a/b). To exclude that inhibition of migration was only due to reduced proliferation, three different assays evaluating cell viability were performed using the same conditions as applied in the scratch assay: MTT assay, neutral red assay, cell count by flow cytometry. Treatment with NVP-AEW541 or lapatinib or their combination did not affect proliferation (Fig. 4c). Only NVP-BEZ235 significantly reduced cell proliferation compared to untreated control (MTT: 82%, neutral red: 87%, cell count: 81%). However, NVP-BEZ235 did not significantly inhibit migration.

Lastly, we extended the study of NVP-AEW541, lapatinib, NVP-BEZ235 in HCC38 and HCC38CisR to the TNBC cell line MDA-MB231. Similarly to the generation of cisplatin-resistant HCC38CisR, we have generated a 2.1-fold more resistant sub-line named MDA-MB231CisR (Additional file 7A, Table 3). Similar to HCC38CisR, MDA-MB231CisR displayed activated EGFR and IGR1R (Additional file 7B). We then tested combinations of dual and triple combinations of kinase inhibitors and cisplatin by MTT assay (Table 3). In accordance with the results obtained in HCC38 and HCC38CisR (Fig. 3a), NVP-BEZ235 had no effect on cisplatin potency in MDA-MB231 but reversed the 2.1-fold cisplatin resistance of MDA-MB231CisR (Table 3). Furthermore, NVP-BEZ235 increased apoptosis induction in combination with cisplatin compared to either compound alone (Additional file 7C). Whereas the combination of NVP-AEW541 plus lapatinib only partially reversed cisplatin resistance in HCC38CisR (Fig. 2g), this combination not only reversed the 2.1-fold resistance of MDA-MB231CisR but shifted cisplatin potency by a factor of 4.8 beyond the sensitivity of MDA-MB231 (Table 3). Notably, similar to the results in HCC38CisR (Fig. 2c), the combination of NVP-AEW541 and lapatinib showed a highly hyper-additive effect in the induction of apoptosis in both MDA-MB231 and MDA-MB231CisR (Additional file 7D).

**Table 3:** IC_50_ values (μM) from MTT assays and corresponding shift factors (SF) of cisplatin alone and after 48 h pretreatment with 1.5 μM NVP-AEW541, 2 μM lapatinib, 20 nM NVP-BEZ235, or 1.5 μM NVP-AEW541 plus 2 μM lapatinib, respectively, in MDA-MB231 and MDA-MB231CisR cells

Compound	MDA-MB231		MDA-MB231 CisR	
	IC_50_	SF	IC_50_	SF
cisplatin	20.9	---	44.0	---
cisplatin + AEW541	22.8	0.9	23.3	1.9
cisplatin + lapatinib	24.1	0.9	24.7	1.8
cisplatin + BEZ235	22.8	0.9	21.8	2.0*
cisplatin + AEW541 + lapatinib	10.1	2.1*	9.16	4.8*

* p < 0.05

Data are mean of 3 experiments

## Discussion

Among breast cancer, TNBC has a poor prognosis due to the lack of targeted hormone or HER2-directed therapy and resistance development against classical cytostatics including cisplatin currently under clinical investigation for TNBC [5]. We have established a cellular model of cisplatin resistance in the TNBC cell line HCC38 to study resistance mechanisms and identify targets for overcoming resistance. The cisplatin resistant cell line labeled HCC38CisR exhibited increased activation observed as phosphorylation of EGFR and IGF1R (Fig. 1). Increased RTK phosphorylation in HCC38CisR was accompanied by faster proliferation (Fig. 1d) and higher susceptibility to EGFR and IGF1R inhibition (Fig. 2a, b) compared to the parental cell line HCC38. By immunostaining, we could demonstrate that an increase in RTK phosphorylation also occurred in HCC38 after short-term (6 h) cisplatin exposure. However, in contrast to HCC38CisR showing a stable cisplatin resistance with permanent EGFR and IGF1R activation, the short-term cisplatin-induced receptor phosphorylation in HCC38 nearly vanished after 1 week of recovery (Fig. 1c). Crosstalk between RTKs as well as the ability of cancer cells to switch between different growth factor receptor pathways is well described [9]. Therefore, lapatinib and NVP-AEW541 were selected to inhibit both activated RTKs in HCC38CisR simultaneously. According to the Cancer Cell Line Encyclopedia [22], EGFR mutations possibly impairing the effect of lapatinib are not described for HCC38. Activation of EGFR and IGF1R was observed in HCC38CisR, but RTK activation is rather associated with than a cause of cisplatin resistance in HCC38CisR as we could not restore cisplatin sensitivity by inhibition of these RTKs with lapatinib and NVP-AEW541 (Fig. 2g) even though both compounds were shown to successfully inhibit EGFR and IGF1R phosphorylation (Fig. 2e, f). Notably, in the highly cisplatin-resistant cell line MDA-MB231CisR (IC_50_ 44.0 μM), we found a 4.8-fold resensitization for cisplatin upon pretreatment with NVP-AEW541 and lapatinib (IC_50_ 9.16 μM, Table 3). We could demonstrate synergy of lapatinib and NVP-AEW541 with respect to inhibition of cell viability (Table 1) and apoptosis induction (Fig. 2c, Additional file 7D). Coincubation with NVP-AEW541 reduced IC_50_ of lapatinib nearly 7-fold (Fig. 1a). This effect may be of clinical importance as the resulting IC_50_ of 0.88 μM is lower than the reported c_max_ of lapatinib (1.7–4 μM) [23, 24]. In cell cycle analysis we could show that the combination of EGFR and IGF1R inhibition resulted in an increase in cells in G_1_ phase. This might be one possible mechanism leading to reduced cell proliferation. These results are in accordance with studies performed on adrenocortical carcinomas applying EGFR and IGF1R inhibitors [25]. Another effect of the combination of lapatinib and NVP-AEW541 in HCC38CisR is the reduction of cell migration (Fig. 4a/b) which was not due to decreased proliferation as shown by simultaneously performed proliferation assays (Fig. 4c). Migration of cancer cells serves as a marker for invasion and the potential to form metastases. As TNBC has a high risk for metastases [26], drugs reducing migration may be valuable in treating TNBC. Although the advantages of combining RTK inhibitors have been shown several years ago [27], the in vitro results have not yet been transferred into clinical benefits [14]. Taking into account that the approach of combining NVP-AEW541 and lapatinib showed only synergy in HCC38CisR but not in HCC38, it might be of value to select tumors according to their RTK activation. Our study demonstrates that the phosphorylation status of RTKs predicts response to the combination of lapatinib and NVP-AEW541 (in HCC38CisR and MDA-MB231CisR) whereas receptor expression showed only marginal differences between non-responding HCC38 and responding HCC38CisR. Therefore, the selection of targeted therapies by receptor phosphorylation rather than receptor expression might be an approach for further studies.

Lapatinib and NVP-AEW541 were ineffective to restore cisplatin sensitivity in HCC38CisR (Fig. 2g). However, Akt was stronger phosphorylated in HCC38CisR than in untreated or short-term (6 h) cisplatin-treated HCC38 (Fig. 1c), assuming an increased activation in the course of cisplatin resistance development. Lapatinib and NVP-AEW541 did not influence downstream Akt phosphorylation (Fig. 2e) suggesting further mechanisms conserving Akt activation [28]. It has been shown that dual inhibition of two kinases in IGF1R signaling pathway is superior to applying only single agents in the TNBC cell line MDA-MB-231 [29]. Therefore, we chose the dual PI3K/mTOR inhibitor NVP-BEZ235 to address increased Akt activation in HCC38CisR. Synergy of NVP-BEZ235 has already been demonstrated for paclitaxel in colon cancer cells [30] and carboplatin in a triple negative breast cancer cell line [31]. Additionally, NVP-BEZ235 has already proven its ability to enhance cisplatin sensitivity in cisplatin resistant bladder cancer cell lines [32].

In our study, NVP-BEZ235 could fully restore cisplatin sensitivity in the cisplatin-resistant TNBC cell line HCC38CisR and acted synergistically with cisplatin (Fig. 3a/d, Table 2). Using KU0063794 and LY2940002, we could demonstrate that it was not sufficient to inhibit mTOR or PI3K alone, respectively, to obtain the NVP-BEZ235-induced effect on cisplatin sensitivity (Fig. 3b). Combining KU0063794 and LY294002 and thereby mimicking the dual inhibition of NVP-BEZ235 increased the effect of each compound alone on cisplatin sensitivity (Fig. 3b). Nevertheless, NVP-BEZ235 was slightly more effective than the combination of KU0063794 and LY294002. Other studies have shown that mTOR inhibition might result in only transient decrease or even increase of phospho-Akt (p-Akt) caused by feedback activation [31, 33]. Thus, these and our results allow the conclusion that the combination of PI3K and mTOR inhibition is preferred over mTOR inhibition alone for cisplatin sensitization. Lastly, synergy between NVP-BEZ235 and cisplatin was not observed in HCC38 even though Akt showed some activation, however lower than in HCC38CisR. This indicates that NVP-BEZ235 enhances cisplatin sensitivity if – next to Akt activation – upstream RTKs such as EGFR and IGF1R are activated. Activated RTKs plus activated Akt may thus serve as potential biomarkers for the use of NVP-BEZ235 in combination with cisplatin in TNBC. These results in HCC38CisR were corroborated by data obtained with MDA-MB231CisR (Table 3, Additional file 7).

## Conclusions

Taken together, activation of EGFR and IGF1R and their downstream signaling pathway kinase Akt is associated with resistance induced by long-term treatment with cisplatin in the TNBC cell line HCC38 and in MDA-MB231. Based on these results, two approaches for treating cisplatin resistant cell lines are presented: 1) Simultaneous inhibition of EGFR and IGF1R by lapatinib and NVP-AEW541 is highly synergistic and results in the induction of apoptosis. Furthermore, co-treatment with lapatinib and NVP-AEW541 may increase cisplatin sensitivity as seen in MDA-MB231CisR. 2) Co-treatment of cisplatin-resistant TNBC cell lines with the PI3K/mTOR inhibitor NVP-BEZ235 and cisplatin is synergistic, fully reversed acquired cisplatin resistance, and may thus constitute a targeted treatment option for cisplatin-resistant TNBC.

## Additional files

Additional file 1: pIC50 values and standard error of the mean. pIC50 values, errors, and IC50 values of all MTT assays performed in this study are listed. (DOC 70 kb)

Additional file 2: cell cycle distribution. The cell cycle distribution in HCC38 after treatment with NVP-AEW541, lapatinib or both compounds is displayed as bar graph. (DOCX 30 kb)

Additional file 3: MTT assay of combination of RTK inhibitors with cisplatin. Influence of 48 h preincubation with 1.5 μM NVP-AEW541, 1 μM lapatinib or a combination of both compounds on cisplatin sensitivity in HCC38. (DOCX 173 kb)

Additional file 4: MTT assay of NVP-BEZ235 . Effect of NVP-BEZ235 on cell viability determined by MTT assay. (DOCX 29 kb)

Additional file 5: Western blot of cleaved PARP upon NVP-BEZ235 and cisplatin treatment. Western Blot on cleaved PARP after treatment of HCC38 with 20 nM NVP-BEZ235 or 2 μM cisplatin or a combination of both compounds. (DOCX 80 kb)

Additional file 6: induction of apoptosis in HCC38 upon NVP-BEZ235 and cisplatin treatment. Induction of apoptotic nuclei in HCC38 after treatment with 2 μM cisplatin, 20 nM NVP-BEZ235 or a combination of both compounds. (DOCX 25 kb)

Additional file 7: Characterization of MDA-MB231 and cisplatin-resistant MDA-MB231CisR. MDA-MB231 and cisplatin-resistant MDA-MB231CisR cells were characterized by MTT assay, phospho-RTK status, and induction of apoptosis upon kinase inhibitor and cisplatin treatment. (DOCX 141 kb)

## Abbreviations

Akt: Protein kinase B
CI: Combination index
c_max_: Maximum serum concentration
EGFR: Human epidermal growth factor receptor
ER: Estrogen receptor
ErbB2: Human epidermal growth factor receptor 2
ErbB3: Human epidermal growth factor receptor 3
ErbB4: Human epidermal growth factor receptor 4
HRP: Horseradish peroxidase
IC_50_: Half-maximum inhibitory concentration
IGF1: Insulin-like growth factor 1
IGF1R: Insulin-like growth factor 1 receptor
mTOR: mechanistic target of rapamycin
MTT: 3-(4,5-Dimethylthiazol-2-yl)-2,5-diphenyltetrazolium bromide
p-Akt: phospho-Akt
PARP: Poly ADP ribose polymerase
p-EGFR: phospho-EGFR
p-ErbB2: phospho-ErbB2
p-ErbB3: phospho-ErbB3
p-ErbB4: phospho-ErbB4
PI3K: Phosphatidylinositol-3-kinase
p-IGF1R: phospho-IGF1R
PR: Progesterone receptor
RTK: Receptor tyrosine kinase
TNBC: Triple negative breast cancer
