# Supplementary material for: Inhibition of PI3K/Akt/mTOR overcomes cisplatin resistance in the triple negative breast cancer cell line HCC38
Source: BMC Cancer. 2017 Nov 3;17:711. doi: 10.1186/s12885-017-3695-5 (PMC5670521; doi:10.1186/s12885-017-3695-5)
Supplement: Supplementary file 1 — pIC50 values and standard error of the mean. pIC50 values, errors, and IC50 values of all MTT assays performed in this study are listed. (DOC 70 kb) [file 12885_2017_3695_MOESM1_ESM.doc]

**Additional file 1**

**pIC_50_ ± SEM**

| Figure 1B |  |  |  |  |
| --- | --- | --- | --- | --- |
|  | pIC_50_ (cisplatin) | IC_50_ (cisplatin) |  |  |
| HCC38 | 5.58 ± 0.02 | 2.7 µM |  |  |
| HCC38CisR | 5.03 ± 0.02 | 9.4 µM |  |  |
|  |  |  |  |  |
| Figure 2 A |  |  |  |  |
|  | pIC_50_  (lapatinib) | IC_50_  (lapatinib) | pIC_50_ (lapatinib) + 1.5 µM AEW541 | IC_50_ (lapatinib)  + 1.5 µM AEW541 |
| HCC38 | 5.04 ± 0.11 | 9.2 µM | 5.05 ± 0.13 | 9.0 µM |
| HCC38CisR | 5.22 ± 0.01 | 6.0 µM | 6.06 ± 0.06 | 0.88 µM |
|  |  |  |  |  |
| Figure 2 B |  |  |  |  |
|  | pIC_50_  (AEW541) | IC_50_  (AEW541) | pIC_50_ (AEW541)  + 2 µM lapatinib | IC_50_ (AEW541)  + 2 µM lapatinib |
| HCC38 | 5.24 ± 0.04 | 5.7 µM | 5.30 ± 0.06 | 5.0 µM |
| HCC38CisR | 5.64 ± 0.03 | 2.3 µM | 5.95 ± 0.03 | 1.1 µM |
|  |  |  |  |  |
| Figure 2 F |  |  |  |  |
|  | pIC_50_ (cisplatin) | IC_50_ (cisplatin) |  |  |
| HCC38 | 5.59 ± 0.02 | 2.6 µM |  |  |
| HCC38CisR | 5.10 ± 0.02 | 7.9 µM |  |  |
| HCC38CisR + 1 µM lapatinib | 5.04 ± 0.02 | 9.1 µM |  |  |
| HCC38CisR + 1.5 µM AEW541 | 5.11 ± 0.02 | 7.8 µM |  |  |
| HCC38CisR + 1.5 µM AEW541 + 1 µM lapatinib | 5.23 ± 0.04 | 5.9 µM |  |  |
|  |  |  |  |  |
| Figure 3 A |  |  |  |  |
|  | pIC_50_ (cisplatin) | IC_50_ (cisplatin) |  |  |
| HCC38 | 5.67 ± 0.02 | 2.1 µM |  |  |
| HCC38 + 20 nM BEZ235 | 5.51 ± 0.05 | 3.1 µM |  |  |
| HCC38CisR | 5.10 ± 0.02 | 7.9 µM |  |  |
| HCC38CisR + 20 nM BEZ235 | 5.71 ± 0.07 | 2.0 µM |  |  |
|  |  |  |  |  |
| Figure 3 B |  |  |  |  |
|  | pIC_50_ (cisplatin) | IC_50_ (cisplatin) |  |  |
| HCC38CisR | 5.10 ± 0.02 | 7.9 µM |  |  |
| HCC38CisR + 5 µM LY294002 | 5.40 ± 0.02 | 4.0 µM |  |  |
| HCC38CisR + 1 µM KU00063794 | 5.45 ± 0.03 | 3.6 µM |  |  |
| HCC38CisR + 1 µM KU00063794 + 5 µM LY294002 | 5.53 ± 0.04 | 2.9 µM |  |  |

| Table 3 |  |  | |  | |  | |
| --- | --- | --- | --- | --- | --- | --- | --- |
|  | MDA-MB231 | | | | MDA-MB231 CisR | | |
|  | pIC_50_ (cisplatin) | | IC_50_ (cisplatin) | | pIC_50_ (cisplatin) | | IC_50_ (cisplatin) |
| cisplatin | 4.68 ± 0.02 | | 20.9 µM | | 4.37 ± 0.03 | | 44.0 µM |
| + AEW541 1.5 µM | 4.64 ± 0.11 | | 22.8 µM | | 4.63 ± 0.08 | | 23.3 µM |
| + BEZ235 20 nM | 4.64 ± 0.09 | | 22.8µM | | 4.66 ± 0.10 | | 21.8 µM |
| + lapatinib 2 µM | 4.62 ± 0.08 | | 24.1 µM | | 4.61 ± 0.06 | | 24.7 µM |
| + 1.5 µM AEW541 + 2 µM lapatinib | 5.00 ± 0.10 | | 10.1 µM | | 5.04 ± 0.13 | | 9.16 µM |
|  |  |  | |  | |  | |
