# Supplementary material for: Inhibition of PI3K/Akt/mTOR overcomes cisplatin resistance in the triple negative breast cancer cell line HCC38
Source: BMC Cancer. 2017 Nov 3;17:711. doi: 10.1186/s12885-017-3695-5 (PMC5670521; doi:10.1186/s12885-017-3695-5)
Supplement: Supplementary file 2 — cell cycle distribution. The cell cycle distribution in HCC38 after treatment with NVP-AEW541, lapatinib or both compounds is displayed as bar graph. (DOCX 30 kb) [file 12885_2017_3695_MOESM2_ESM.docx]

**Additional file 2**


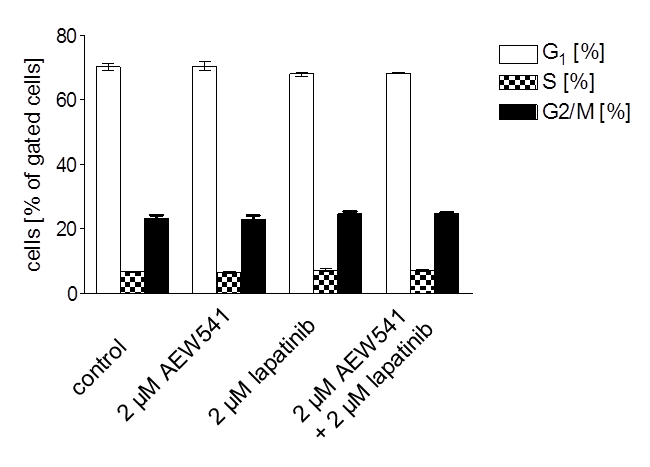


**Cell cycle distribution in HCC38 after treatment with NVP-AEW541, lapatinib or both compounds.**
