# Supplementary material for: Inhibition of PI3K/Akt/mTOR overcomes cisplatin resistance in the triple negative breast cancer cell line HCC38
Source: BMC Cancer. 2017 Nov 3;17:711. doi: 10.1186/s12885-017-3695-5 (PMC5670521; doi:10.1186/s12885-017-3695-5)
Supplement: Supplementary file 3 — MTT assay of combination of RTK inhibitors with cisplatin. Influence of 48 h preincubation with 1.5 μM NVP-AEW541, 1 μM lapatinib or a combination of both compounds on cisplatin sensitivity in HCC38. (DOCX 173 kb) [file 12885_2017_3695_MOESM3_ESM.docx]

**Additional file 3**

**
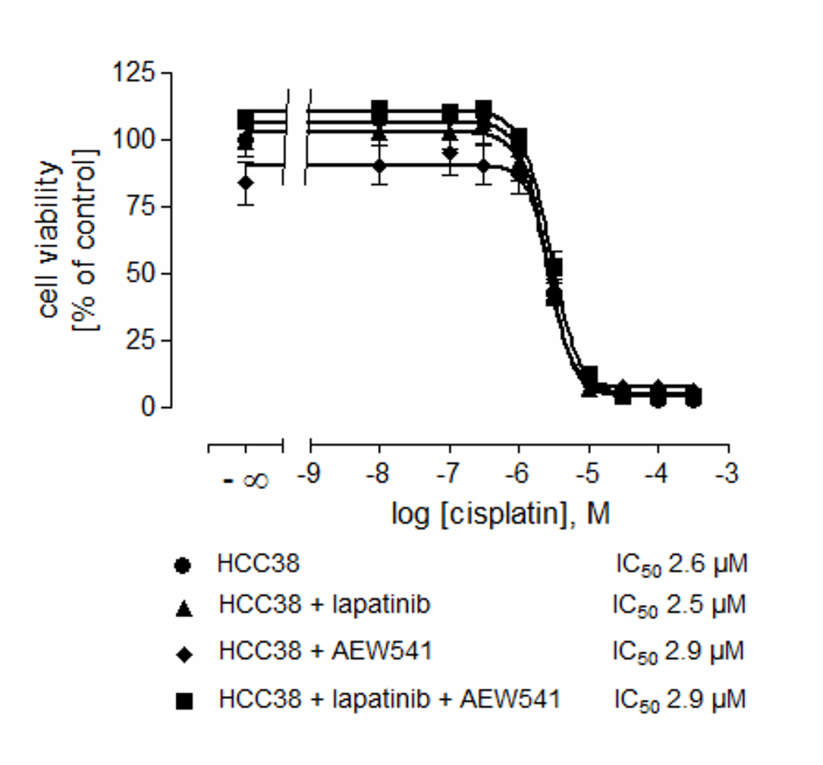
**

**Influence of 48 h preincubation with 1.5 µM NVP-AEW541, 1 µM lapatinib or a combination of both compounds on cisplatin sensitivity in HCC38.**
