# Supplementary material for: Inhibition of PI3K/Akt/mTOR overcomes cisplatin resistance in the triple negative breast cancer cell line HCC38
Source: BMC Cancer. 2017 Nov 3;17:711. doi: 10.1186/s12885-017-3695-5 (PMC5670521; doi:10.1186/s12885-017-3695-5)
Supplement: Supplementary file 4 — MTT assay of NVP-BEZ235 . Effect of NVP-BEZ235 on cell viability determined by MTT assay. (DOCX 29 kb) [file 12885_2017_3695_MOESM4_ESM.docx]

**Additional file 4**

**Effect of NVP-BEZ235 on cell viability determined by MTT assay.**
