# Supplementary material for: Inhibition of PI3K/Akt/mTOR overcomes cisplatin resistance in the triple negative breast cancer cell line HCC38
Source: BMC Cancer. 2017 Nov 3;17:711. doi: 10.1186/s12885-017-3695-5 (PMC5670521; doi:10.1186/s12885-017-3695-5)
Supplement: Supplementary file 5 — Western blot of cleaved PARP upon NVP-BEZ235 and cisplatin treatment. Western Blot on cleaved PARP after treatment of HCC38 with 20 nM NVP-BEZ235 or 2 μM cisplatin or a combination of both compounds. (DOCX 80 kb) [file 12885_2017_3695_MOESM5_ESM.docx]

**Additional file 5**

**
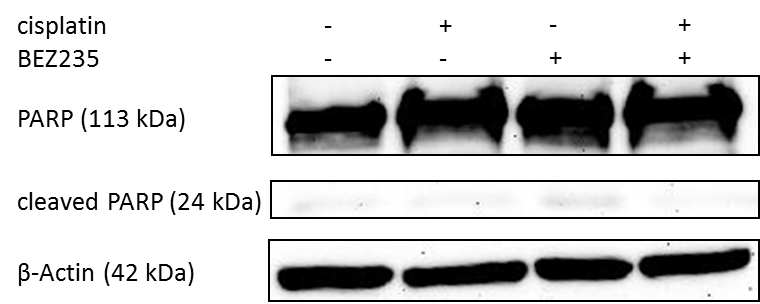
**

**Western Blot on cleaved PARP after treatment of HCC38 with 20 nM NVP-BEZ235 or 2 µM cisplatin or a combination of both compounds.**
