# Supplementary material for: Inhibition of PI3K/Akt/mTOR overcomes cisplatin resistance in the triple negative breast cancer cell line HCC38
Source: BMC Cancer. 2017 Nov 3;17:711. doi: 10.1186/s12885-017-3695-5 (PMC5670521; doi:10.1186/s12885-017-3695-5)
Supplement: Supplementary file 6 — induction of apoptosis in HCC38 upon NVP-BEZ235 and cisplatin treatment. Induction of apoptotic nuclei in HCC38 after treatment with 2 μM cisplatin, 20 nM NVP-BEZ235 or a combination of both compounds. (DOCX 25 kb) [file 12885_2017_3695_MOESM6_ESM.docx]

**Additional file 6**

**
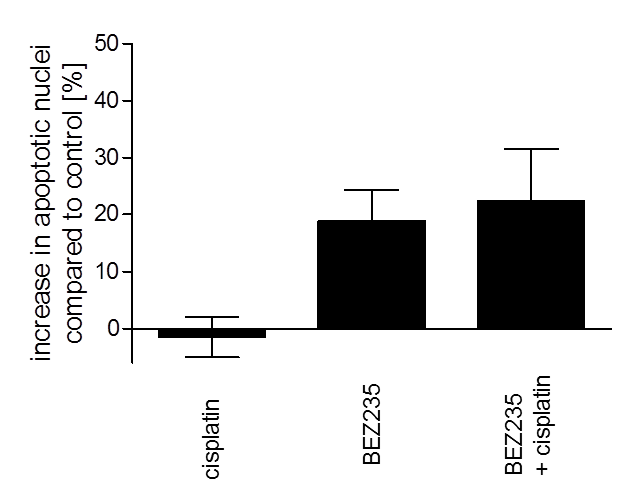
**

**Induction of apoptotic nuclei in HCC38 after treatment with 2 µM cisplatin, 20 nM NVP-BEZ235 or a combination of both compounds.**
