# Supplementary material for: Inhibition of PI3K/Akt/mTOR overcomes cisplatin resistance in the triple negative breast cancer cell line HCC38
Source: BMC Cancer. 2017 Nov 3;17:711. doi: 10.1186/s12885-017-3695-5 (PMC5670521; doi:10.1186/s12885-017-3695-5)
Supplement: Supplementary file 7 — Characterization of MDA-MB231 and cisplatin-resistant MDA-MB231CisR. MDA-MB231 and cisplatin-resistant MDA-MB231CisR cells were characterized by MTT assay, phospho-RTK status, and induction of apoptosis upon kinase inhibitor and cisplatin treatment. (DOCX 141 kb) [file 12885_2017_3695_MOESM7_ESM.docx]

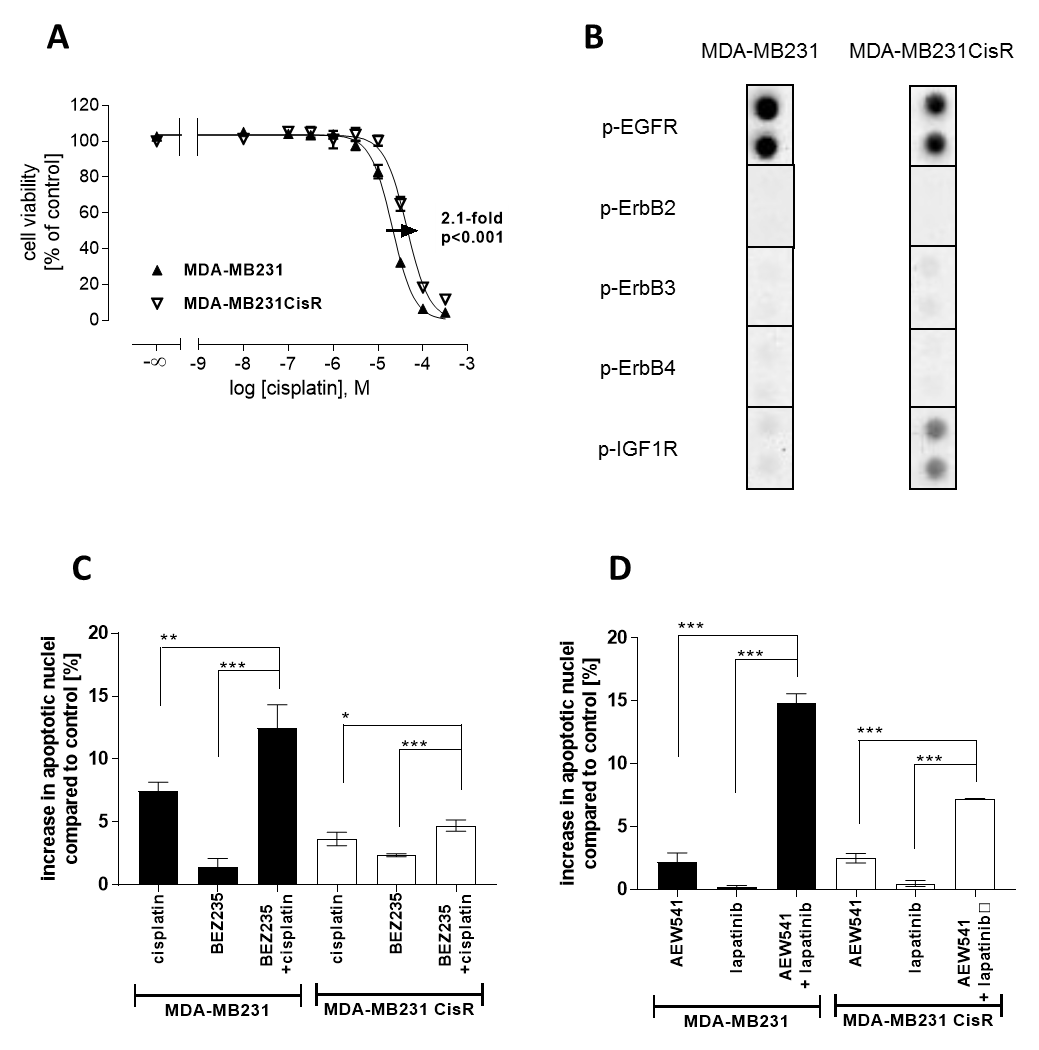


**Additional file 7**

**Characterization of MDA-MB231 and cisplatin-resistant MDA-MB231CisR. (A)**  Weekly exposure of MDA-MB231 with the IC_50_ of cisplatin for 6h resulted in the cisplatin resistant subclone MDA-MB231CisR with a resistance factor of 2.1 (p<0.001). IC_50_ cisplatin MDA-MB-231: 20.9 µM; IC_50_ cisplatin MDA-MB231CisR: 44.0 µM. Shown are mean +/- SEM, n = 3. **(B)** Detail of phospho-RTK-array displays phosphorylation status of EGFR-family and IGF1R in MDA-MB231 and MDA-MB231CisR. **(C)** Induction of apoptosis by NVP-BEZ235 and cisplatin. 20 nM NVP-BEZ235 was incubated 24h prior to addition of 20 µM (MDA-MB231) or 50 µM cisplatin (MDA-MB231CisR) for 6h followed by 24h of recovery. Combination of NVP-BEZ235 with cisplatin increased apoptotic nuclei compared to cisplatin alone or NVP-BEZ235 alone (*p < 0.05, **p < 0.01, ***p < 0.001). **(D)** In MDA-MB231 and MDA-MB231 CisR, the combination of NVP-AEW541 and lapatinib significantly induced apoptosis in a hyper-additive manner (***p<0.001). NVP-AEW541 and lapatinib were used at 2 µM. Cells were treated for 48h and the amount of apoptotic nuclei in the control was subtracted from treated samples (***p < 0.001).
